# Impaired chronic pain‐like behaviour and altered opioidergic system in the TASTPM mouse model of Alzheimer's disease

**DOI:** 10.1002/ejp.1288

**Published:** 2018-07-30

**Authors:** Y. Aman, T. Pitcher, C. Ballard, M. Malcangio

**Affiliations:** ^1^ Wolfson Centre for Age Related Diseases King's College London UK; ^2^ Medical School University of Exeter UK

## Abstract

**Background:**

Chronic pain conditions, especially osteoarthritis (OA), are as common in individuals with Alzheimer's disease (AD) as in the general elderly population, which results in detrimental impact on patient's quality of life. However, alteration in perception of pain in AD coupled with deteriorating ability to communicate pain sensations often result in under‐diagnosis and inappropriate management of pain. Therefore, a better understanding of mechanisms in chronic pain processing in AD is needed. Here, we explored the development and progression of OA pain and the effect of analgesics in a transgenic mouse model of AD.

**Methods:**

Unilateral OA pain was induced chemically, via an intra‐articular injection of monosodium iodoacetate (MIA) in the left knee joint of AD‐mice (TASTPM) and age‐ and gender‐matched C57BL/6J (WT). Pharmacological and biochemical assessments were conducted in plasma and spinal cord tissue.

**Results:**

MIA resulted in hind paw mechanical hypersensitivity (allodynia), initiating on day 3, in TASTPM and WT controls. However, from 14 to 28 days, TASTPM displayed partial attenuation of allodynia and diminished spinal microglial response compared to WT controls. Naloxone, an opioid antagonist, re‐established allodynia levels as observed in the WT group. Morphine, an opioid agonist, induced heightened analgesia in AD‐mice whilst gabapentin was devoid of efficacy. TASTPM exhibited elevated plasma level of β‐endorphin post‐MIA which correlated with impaired allodynia.

**Conclusions:**

These results indicate an alteration of the opioidergic system in TASTPM as possible mechanisms underlying impaired persistent pain sensitivity in AD. This work provides basis for re‐evaluation of opioid analgesic use for management of pain in AD.

**Significance:**

This study shows attenuated pain‐like behaviour in a transgenic mouse model of Alzheimer's disease due to alterations in the opioidergic system and central plasticity mechanisms of persistent pain.

## 1 Introduction

Alzheimer's disease (AD) is the most common cause of dementia (>60% of dementia) in the elderly population. Clinically, it is characterized by a global cognitive deficit ranging from loss of memory to impaired judgement and reasoning (Tanzi and Bertram, 2001). Experience of pain is a key contributor to challenge of care in AD individuals and is often associated with age‐related medical comorbidities, commonly musculoskeletal conditions such as osteoarthritis (OA).

OA is the most common age‐related musculoskeletal condition affecting the elderly population over the age of 50 (Litwic et al., 2013). Clinical symptoms are primarily chronic pain coupled with joint stiffness and dysfunction (Hunter et al., 2008). Due to an incomplete understanding of the underlying mechanisms, there are no cure or disease modifying drugs, at present. Currently, the best treatment options are physiotherapy, pain relief (i.e. paracetamol, non‐steroidal anti‐inflammatory drugs (NSAIDs), and opiates) and surgical joint replacement (Hunter and Felson, 2006).

The cause and incidence of chronic pain conditions, in particular OA, are as common in individuals with AD as in the general elderly population. Despite availability of pain treatment options, assessment and treatment of pain in AD is often difficult, which has a negative impact on the quality of life (Corbett et al., 2012). Under‐treated and inappropriate prescribing in this patient group results in reduced mobility, muscle weakness and falls, which consequently has a major detrimental impact on quality of life and is a key contributor to the presentation of neuropsychiatric symptoms such as aggression and mood disorders (Ballard et al., 2014; Rajkumar et al., 2017).

It remains unclear whether differences observed in reporting and management of pain are a result of impaired memory and ability to communicate; and/or the perception of pain is altered due to progressive degeneration of cortical and sub‐cortical regions involved in processing and transmission of nociceptive information (Hyman et al., 1984; Scherder and Bouma, 2000). AD‐associated neuropathological hallmarks, namely, extracellular β‐amyloid (Aβ) plaques and intracellular neurofibrillary tangles, accompanied by neuroinflammation have been detected in regions involved in pain processing, namely the spinal cord and the thalamus (Schmidt et al., 2001; Rub et al., 2002; Aman et al., 2016). To date, difficulties in assessment of pain in individuals with cognitive impairments has been identified; however, there is a lack of understanding of underlying mechanisms of pain in this susceptible population (Ballard et al., 2009; Corbett et al., 2012).

Therefore, a better understanding of the pathophysiological mechanisms underlying development and progression of OA is essential for improving the clinical management of this chronic pain condition in patients with AD. Here, we assessed development of chemically induced OA pain, via an intra‐articular administration of monosodium iodoacetate (MIA), in the double‐mutant TASTPM transgenic mouse model of AD (Howlett et al., 2004; Ogbonna et al., 2013). The TASTPM transgenic mouse strain carries mutant versions of the amyloid precursor protein (APPswe) and presenilin‐1 (PS1.M146V) associated with familial forms of AD (Howlett et al., 2004). Histological and behavioural analysis of these animals has identified pathological characteristics and cognitive alterations from 3 and 6 months of age, respectively, that reflect some aspects of the pathological changes and cognitive defects observed in people with AD (Howlett et al., 2004, 2008). Specifically, we characterized pain‐like behaviour and associated pathological changes in the periphery and the spinal cord. In addition, we evaluated the effectiveness of commonly used analgesics in alleviating MIA‐induced nociceptive hypersensitivity in TASTPM and age‐ and gender‐matched non‐transgenic controls.

## 2 Materials and methods

### 2.1 Animals

Experiments were performed on 6–8 months old adult male and female heterozygous double‐mutant TASTPM transgenic mouse model of AD obtained from GlaxoSmithKline (GSK) (Aman et al., 2016). The TASTPM were generated using TAS10 transgenic mice expressing the Swedish mutant human amyloid precursor protein (APP) (695‐aa isoform) under the control of the murine Thy‐1 promoter and transgenic mice over expressing presenilin‐1 M146V mutation (TPM) driven through the murine Thy‐1 promoter (Richardson et al., 2003; Howlett et al., 2004). Briefly, TAS10 (Thy‐1.APPswe) mice were generated and backcrossed onto a pure C57BL/6 background before being crossed with TPM (Thy‐1.PSEN‐1.M146V) mice to produce heterozygous double‐mutant TASTPM mice. Age‐ and gender‐matched C57BL/6J (WT) obtained from Charles River were used as controls. All animals were housed in the Biological Services Unit, King's College London; maintained in 12 h day/night cycle with *ad libitum* access to food and water; and were allowed acclimatization for 7 days prior to behavioural experiments. All experiments were conducted in accordance with United Kingdom Home Office Regulations (Animal Scientific Procedures Act, 1986). Experimental study groups were randomized, and experiments were performed by an observer unaware of treatments.

### 2.2 Monosodium iodoacetate (MIA) model of osteoarthritis pain

Unilateral knee joint osteoarthritis was induced by a single intra‐articular injection of 10 μL sterile 0.9% saline containing 1 mg MIA (Sigma, UK) into the left knee using a 28G needle and a Hamilton syringe, under isoflurane/O_2_ inhalation anaesthesia (Ogbonna et al., 2013; Pitcher et al., 2016). Control mice received an intra‐articular injection of sterile saline (10 μL).

### 2.3 Behavioural testing

Weight bearing changes were assessed as a measure of ongoing primary pain associated hypersensitivity. Weight bearing was assessed using an incapacitance tester (Linton Instrumentation, UK) as previously described (Ogbonna et al., 2013). Briefly, mice were placed in a Perspex enclosure so that each hind paw is rested on separate transducer pads. Once the mice were settled and in correct position, the force exerted by each hind limb was measured over a period of 1 s. The first three sets of 1 s measurements were taken and then averaged. These values were then transformed to give the percentage of total hind limb weight borne on the ipsilateral side using the formula:

$$\begin{array}{cc} & \text{Weight Borne}\\ & =\frac{\text{Ipsilateral weight borne}\times 100}{\text{Ipsilteral weight borne}+\text{Contralateral weight borne}} \end{array}$$

A value of 50% represents equal weight distributed across both hind limbs, whereas a value of less than 50% was indicative of reduced weight borne on the ipsilateral hind limb.

Mechanical withdrawal thresholds were assessed by calibrated von Frey monofilaments (0.007–1.00 g) application to the plantar surface of the hind paw. The 50% paw withdrawal threshold (PWT) was determined according to the ‘up‐down’ method (Aman et al., 2016).

Behaviour was assessed prior to (baseline observations) and at regular intervals: 3, 7, 10, 14, 17, 21, 24 and 28 days following MIA/saline injection.

### 2.4 Analgesic testing

Celecoxib (30 mg/kg) was purchased from Sigma and dissolved in 1% (w/v) methyl cellulose solution. Paracetamol (acetaminophen; 300 mg/kg) and morphine sulfate (6 mg/kg) were obtained from Sigma and were dissolved in sterile saline. Gabapentin (60 mg/kg) was supplied by LKT Laboratories inc. and was dissolved in dH_2_O. Drugs were administrated either subcutaneously (s.c.: morphine) or orally (p.o.: celecoxib, paracetamol and gabapentin). Celecoxib was administrated up to day 7; and paracetamol, morphine and gabapentin were delivered between days 10 to 28 after MIA injection in a different set of animals. On the day of testing, pre‐dose behavioural readings were recorded and the effect of the drug‐administrated (post‐dose) mechanical thresholds was monitored at 30 min (paracetamol), 1 h (celecoxib and morphine) or 3 h (gabapentin). Data for drug effects were expressed as mean grams threshold, calculated as post‐dose–pre‐dose value.

### 2.5 Naloxone administration

Naloxone hydrochloride (1 mg/kg) was obtained from Sigma and dissolved in sterile saline. Naloxone hydrochloride was administered via an intra‐peritoneal (i.p.) injection on day 24 post‐MIA injection. On the day of testing, pre‐dose behavioural readings were recorded and the effect of the drugs administrated was monitored over a period of 3 h, with mechanical hypersensitivity tests carried out at 30, 90 and 180 min after administration.

### 2.6 Perfusion

Four weeks after MIA injection, mice were terminally anaesthetized with an overdose of sodium pentobarbital (~150 mg/kg body weight, Euthatal, Merial Animal Health, UK) and perfused transcardially with heparinized (1 U/mL) sterile saline followed by 4% paraformaldehyde (PFA, Sigma,) fixative solution containing 1.5% picric acid (Sigma) in phosphate buffer (PB, 0.1 mol/L, pH 7.4).

### 2.7 Knee histology

The knees were dissected out, and the surrounding muscle was trimmed. Tissues were post‐fixed 72 h in 4% PFA, placed in decalcifying solution containing 10% (w/v) formic acid (VWR, UK) for 72 h, embedded in paraffin and sagittal sections (6 μm) were cut using a microtome that was mounted onto Superfrost Plus microscope slides (BDH, UK). Sections were de‐waxed in xylene, rehydrated in ethanol, followed by washing in dH_2_O before staining with either in 0.05% (v/v) toluidine blue dye (pH 4, Sigma) or Gill's (2) haematoxylin and eosin (H&E, Sigma). Sections were subsequently rapidly dehydrated, cleared in xylene and coverslipped using dibutylphthalate in xylene mounting medium (DPX, Sigma). The knee joint histology was visualized using Zeiss microscope (Axioskop) and images were captured using Zeiss Axiocam MRc and the software Axiovision Release 4.6.

### 2.8 Immunohistochemistry

Spinal cords (L3–L6) were removed and immersion‐fixed in 4% PFA fixative solution containing 1.5% picric acid for 24 h at 4 °C. Subsequently, spinal cords were cryoprotected in a solution of 20% sucrose in PB at 4 °C for at least 48 h and subsequently embedded in optimum cutting temperature (OCT, BDH, UK) medium and then snap‐frozen using liquid nitrogen and stored at −80 °C. Transverse spinal cord sections were cut (20‐μm thick) using a cryostat and thaw mounted onto Superfrost Plus microscope slides.

Immunofluorescence was conducted on frozen slide‐mounted 20‐μm transverse spinal cord sections. Briefly, sections were blocked in PBS with 1% (w/v) bovine serum albumin (BSA, Sigma), 0.2% (w/v) sodium azide (Sigma) and 0.1% Triton X‐100 (BDH, UK) prior to an overnight incubation with the primary antibody rabbit anti‐phospho‐p38 (p‐p38, 1:100, Cell Signaling). Sections were then incubated for 1 h with biotinylated donkey anti‐rabbit (1:400; Jackson ImmunoResearch) followed by peroxidase containing avidin–biotin complex (Vectastatin^®^ ABC Kit, Vector) and bitotinylated tyramide (NEN, Life Science Products), which was detected with Extra‐Avidin‐conjugated FITC (1:500, Sigma). Sections were then incubated overnight with rabbit anti‐ionized calcium binding adaptor molecule 1 (IBA1, 1:1000, Wako) followed by Alexa Fluor 546‐conjugated donkey anti‐rabbit antibody (1:1000, Molecular Probes). Sections were then coverslipped with Vectashield Mounting Medium containing nuclear marker 4′,6‐diamidino‐2‐phenylindole·2HCl (DAPI; Vector Laboratories, UK). All steps were conducted at room temperature and all antibody solutions were prepared in PBS with 1% BSA, 0.1% Triton X‐100 and 0.2% sodium azide. The specificity of immunoreactivity was confirmed by omitting the primary antibody; and the immunofluorescent staining was visualized using Zeiss microscope (Imager.Z1) and images were captured using Zeiss AxioCam MRm and the software Axiovision Release 4.8.2. (Zeiss, UK).

### 2.9 Quantitative assessment of immunostaining

Quantitative assessment of number of IBA1 immunopositive microglial cells that also expressed p‐p38 immunoreactivity in the spinal cord were calculated by counting the frequency of IBA1 immunopositive cells in a defined region of 4 × 10^4^ μm^2^ placed onto the superficial laminae of the dorsal horn (laminae I–III) using Axiovision LE 4.8 software. Three L3–L5 sections at least 200 μm apart from each animal were randomly selected from at least four animals per experimental group.

### 2.10 Enzyme immunoassay (EIA)

Four weeks after MIA injection, mice were terminally anaesthetized with an overdose of sodium pentobarbital (~150 mg/kg body weight) and using a 25G needle attached to 1‐mL syringe a cardiac puncture was performed and blood was drawn into heparin‐coated beads containing tubes. Samples were immediately placed on ice for half an hour prior centrifuging at 13,000 rpm for 20 min at 4 °C and the supernatant (plasma) was collected, which was assayed for β‐endorphin content by EIA using the endorphin, beta (mouse, bovine, ovine, camel) – EIA kit (Phoenix Pharmaceuticals). The peptide standards (0.01–100 ng/mL), assay buffer (total binding) or positive control were run in duplicate according to the manufacturer's protocol. The optical density of each well was determined at a wavelength of 450 nm.

### 2.11 Statistical analysis

The data were analysed using SigmaPlot 12.5 (Systat Software, San Jose, CA). The statistical tests performed and the numbers of animals used are displayed in the results section and within the figure legends. Where data were not normally distributed, the appropriate non‐parametric test was applied. Graphs were generated using GraphPad Prism 5 (Graphpad Software Inc., San Diego, USA). All data are presented as mean ± standard error mean (SEM) and a probability value less than 0.05 (*p* < 0.05) was considered statistically significant.

## 3 Results

### 3.1 Weight asymmetry absent in TASTPM following MIA injection

**Figure 1:**
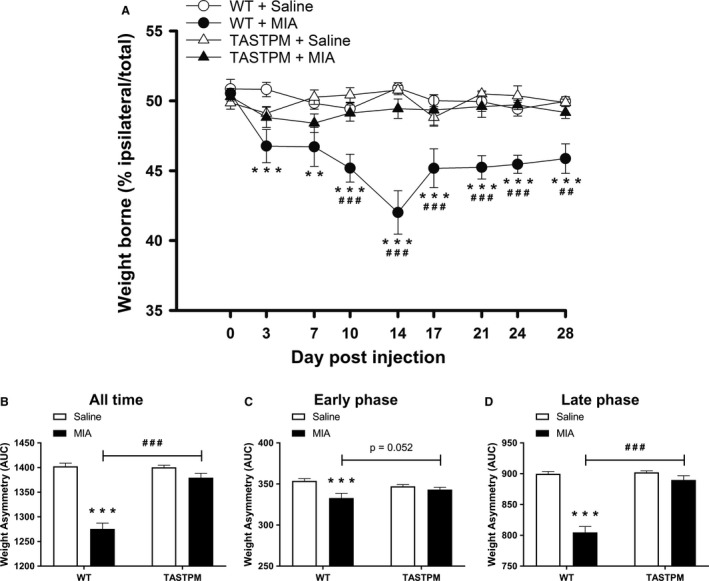
MIA‐induced weight asymmetry absent in TASTPM mice. Weight borne on ipsilateral hind limb, calculated as percentage of ipsilateral weight borne/total weight borne (ipsilateral + contralateral weight borne), by 6 months old TASTPM and age‐ and gender‐matched wild‐type (WT), was monitored at regular intervals prior to (baseline, 0) and up to 28 days post‐intra‐articular administration of MIA (1 mg/10 μL) or saline (control) into the left knee joint (A). Alteration in weight distribution was compared between experimental groups within genotype (**p < 0.01, ***p < 0.001) and between genotype within experimental group (^##^ *p* < 0.01, ^###^ *p* < 0.001) was detected using two‐way repeated measures ANOVA followed by the Student–Newman–Keuls post hoc test. The number of male and female per experimental group: WT + Saline (*n* = 10: five males and five females); WT + MIA (*n* = 8: four males and four females); TASTPM + Saline (*n* = 8: four males and four females); and TASTPM + MIA (*n* = 10: five males and five females). As no difference in thresholds was observed, prior to or at regular intervals post‐MIA administration, between male and female in both WT and TASTPM mice, the data were pooled. Data are as mean weight borne ± SEM (*n* = 8–10 mice per experimental group). Area under the curve (AUC) analysis was calculated and expressed as weight asymmetry where a lower value represents more severe weight asymmetry. All time is the analysis of AUC for baseline – day 28 (B), early phase (baseline – day 7) (C) and late phase (day 10 – day 28) (D). Statistical comparisons between experimental groups within genotype (***p < 0.001) and between genotype within experimental group (^###^ *p* < 0.001) were conducted using two‐way ANOVA followed by the Student–Newman–Keuls post hoc test. All values are expressed as mean ± SEM (*n* = 8–10 mice per experimental group).

As previously reported (Ogbonna et al., 2013), WT mice injected with intra‐articular MIA displayed significant reduced weight borne on the ipsilateral hind limb compared to their respective saline control on day 3 which remains consistently different up till day 28. In contrast, TASTPM mice injected with MIA did not exhibit any significant weight asymmetry compared to the saline‐injected TASTPM group throughout the course of the study (Fig. 1A). An area under the curve (AUC) analysis revealed significantly reduced weight borne on the ipsilateral hind limb by only MIA‐injected WT mice compared to their respective saline group during both the early (day 0–day 7) and late (day 10–day 28) phases (Fig. 1B–D). Thus, weight asymmetry was observed to be absent in the TASTPM mouse model of AD following an intra‐articular injection of MIA.

### 3.2 Impaired maintenance of MIA‐induced mechanical hypersensitivity in TASTPM mice

**Figure 2:**
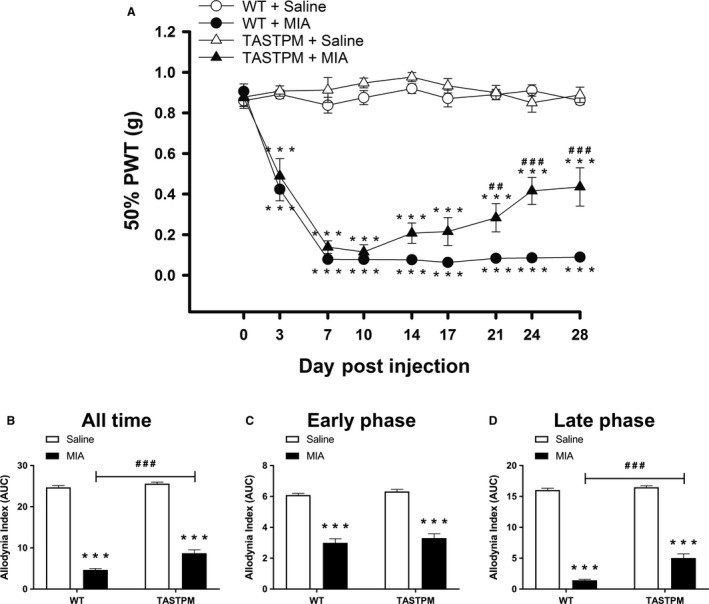
Partial reversal of MIA‐induced mechanical hypersensitivity in the ipsilateral hind paw of TASTPM mice. A representation of mechanical withdrawal responses in the ipsilateral hind paw of 6 months old TASTPM and age‐ and gender‐matched wild‐type (WT) that were measured at regular intervals prior to (baseline, 0) and up to 28 days post‐intra‐articular administration of MIA (1 mg/10 μL) or saline (control) into the left knee joint (A). Alteration in behaviour compared between experimental groups (MIA vs. Saline) within genotype (WT or TASTPM) (***p < 0.001) and between genotype (TASTPM vs. WT) within experimental group (Saline or MIA) (^##^ *p* < 0.01, ^###^ *p* < 0.001) was detected using two‐way repeated measures analysis of variance (ANOVA) followed by the Student–Newman–Keuls post hoc test. The number of male and female per experimental group: WT + Saline (*n* = 10: five males and five females); WT + MIA (*n* = 8: four males and four females); TASTPM + Saline (*n* = 8: four males and four females); and TASTPM + MIA (*n* = 10: five males and five females). As no difference in thresholds was observed, prior to or at regular intervals post‐MIA administration, between male and female in both WT and TASTPM mice, the data were pooled. Data are expressed as mean 50% paw withdrawal threshold (PWT) ± SEM (*n* = 8–10 mice per experimental group). Area under the curve (AUC) for ipsilateral hind paw was calculated and expressed as allodynia index where a lower value represents more severe mechanical hypersensitivity. All time is the analysis of AUC for baseline – day 28 (B), early phase (baseline – day 7) (C) and late phase (day 10 – day 28) (D). Statistical comparisons between experimental groups within genotype (***p < 0.001) and between genotype within experimental group (^###^ *p* < 0.001) were observed using two‐way ANOVA followed by the Student–Newman–Keuls post hoc test. All values are expressed as mean AUC ± SEM (*n* = 8–10 mice per experimental group).

MIA injection into the mouse knee is associated with referred mechanical hypersensitivity in the ipsilateral hind paw (Ogbonna et al., 2013). Here, this observation was confirmed in WT mice with a significant reduction in mechanical withdrawal thresholds initiating on day 3, peaking on day 7 and lasting up to day 28 as compared to respective saline controls (Fig. 2A). We observed that mechanical hypersensitivity developed comparably in TASTPM; however, the AD mice exhibited a partial recovery demonstrated by significantly higher mechanical thresholds than in WT mice from day 21 post‐MIA injection (Fig. 2A). Analysis of AUC demonstrated the mechanical withdrawal thresholds of MIA‐injected TASTPM mice were higher than WT thresholds, in particular during the late phase between days 10 to 28 (Fig. 2B–D).

These data demonstrate that the extent of MIA‐induced pain‐like behaviour is attenuated in TASTPM AD mice. Specifically, TASTPM fail to maintain late phase of mechanical hypersensitivity which is postulated to represent the established phase of the model.

### 3.3 MIA‐induced cartilage degradation

**Figure 3:**
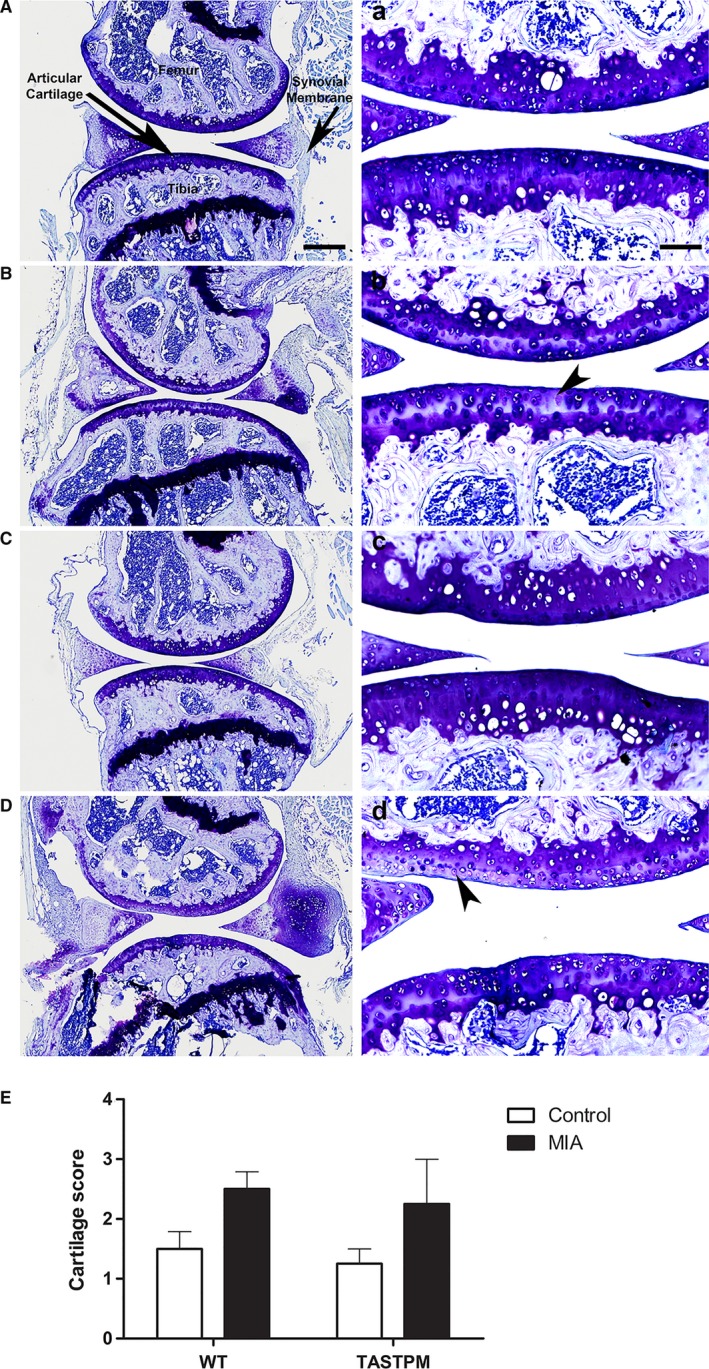
MIA‐induced knee cartilage degradation. Representation of toluidine blue staining of articular cartilage from control and MIA‐injected (ipsilateral) knee joints of wild‐type (WT, A–B) and TASTPM (C–D) mice 28 days post‐MIA. High magnification images for each experimental group representing the outlined box (a–d). Intact articular cartilage with regular and intense staining of proteoglycans was observed in the control WT and TASTPM mice, whereas 28 days post‐MIA injection, degradation and loss of ipsilateral articular cartilage (arrowheads) were induced in both WT and TASTPM knee joints. The scale bar represents 200 μm (A–D) and 100 μm (a–d). Quantitative analysis of knee joint pathology 28 days post‐MIA injection revealed an increase, yet insignificant (*p* > 0.05 Student's *t*‐test), in cartilage degradation exhibited by both TASTPM and WT compared to their respective saline controls (E). Data are shown as mean ± SEM (*n* = 4 (two males and two females) per experimental group).

Histological examination of toluidine blue staining of knee joints 28 days following intra‐articular injections demonstrates that saline‐treated knees from both WT and TASTPM mice have intact and smooth articular surfaces (Fig. 3A and C). MIA‐treated knees from WT and TASTPM mice displayed thinning cartilage and loss of proteoglycan staining (Fig. 3B and D; shown with arrowheads). Quantitative analysis revealed trends of increased knee joint cartilage degradation (not significant, *p* > 0.05) in both TASTPM and WT mice 4 weeks post‐MIA administration compared to their respective controls (Fig. 3E).

### 3.4 MIA‐induced synovial inflammation

**Figure 4:**
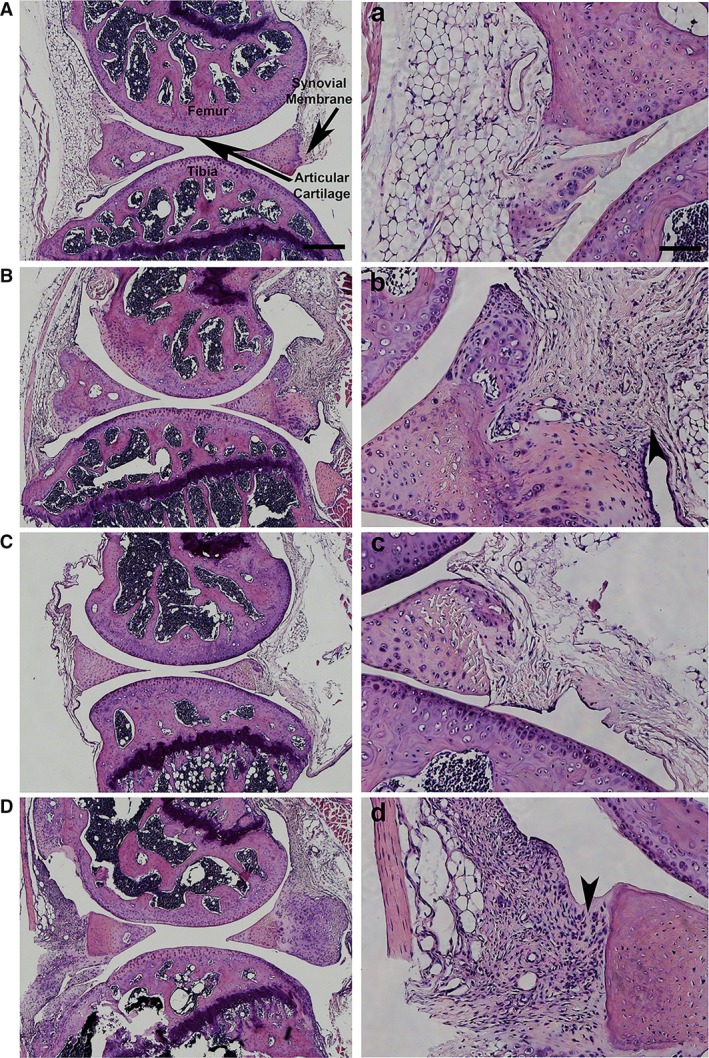
MIA‐induced inflammation in the synovial membrane. Representation of haematoxylin and eosin staining (H&E) of articular cartilage from control and MIA‐injected (ipsilateral) knee joints of wild‐type (WT, A–B) and TASTPM (C–D) mice 28 days post‐MIA. High magnification images for each experimental group representing the outlined box (a–d). H&E staining displayed minimal nuclei within the synovial membrane of WT and TASTPM controls. However, 28 days post‐MIA resulted in the thickening of the synovial membrane lining and infiltration of cells in the ipsilateral synovial membrane of MIA‐injected animals only (arrowheads). The scale bar represents 200 μm (A–D) and 100 μm (a–d).

Microscopic examination of the knee joint using H&E, 28 days post‐MIA injection, demonstrated sparse distribution of haematoxylin‐positive nuclei within the synovial membrane of saline‐treated knees from both WT and TASTPM mice (Fig. 4A and C). MIA‐treated knees from WT and TASTPM mice displayed thickening of the synovial membrane and abundant haematoxylin‐positive cell nuclei within the synovial membrane (Fig. 4B and D).

### 3.5 Attenuated MIA‐induced spinal microgliosis in TASTPM mice

**Figure 5:**
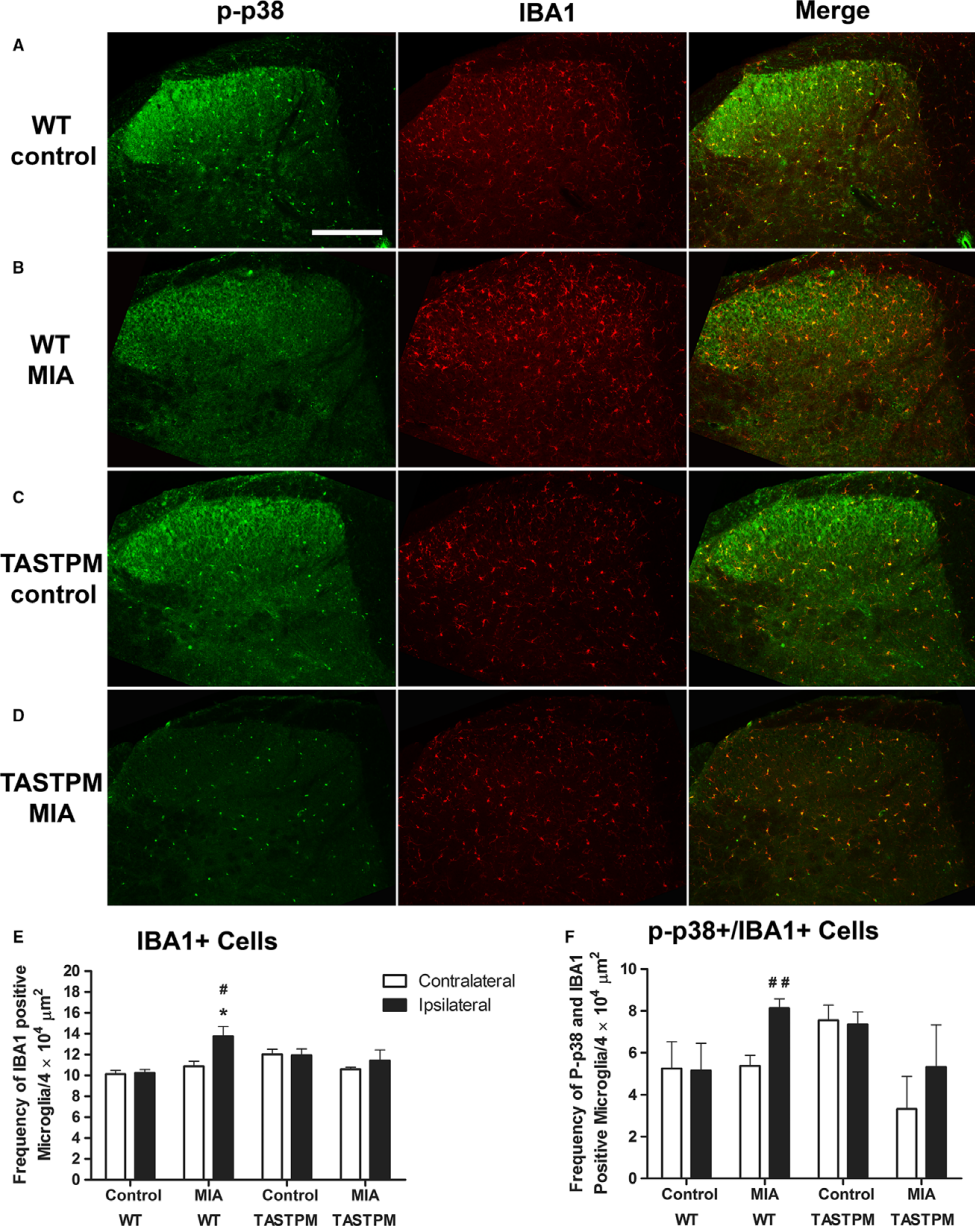
Lack of MIA‐induced spinal microgliosis exhibited by TASTPM mice. Representative images showing phospho‐p38 (p‐p38) expressing microglia (IBA1) in the dorsal horn of the spinal cord (L4–L5) 28 days after intra‐articular injection of saline (control) or MIA of TASTPM and age‐ and gender‐matched wild‐type (WT) mice (A–D). The scale bar represents 200 μm. Quantification of IBA1 immunopositive cells (E) and P‐p38 expressing IBA1 cell frequency (F) was conducted in pooled L4 and L5 dorsal horn of MIA and control mice. WT mice exhibited significantly greater frequency of IBA1 immunopositive microglia in the ipsilateral dorsal horn compared to WT control ipsilateral (*p < 0.05, Student's *t*‐test) and WT MIA contralateral, where increase in activated microglia was also evident (^#^ *p* < 0.05, ^##^ *p* < 0.01, Student's *t*‐test). Contrastingly, no apparent changes in microglial or activated microglia were observable in the ipsilateral MIA‐injected TASTPM mice compared to neither TASTPM control ipsilateral nor TASTPM MIA contralateral (*p* > 0.05, Student's *t*‐test). Data are shown as mean ± SEM (*n* = 4 (2 males and 2 females) per experimental group).

Spinal changes associated with intra‐articular injection of MIA include a microglial response in the ipsilateral dorsal horn associated with an increased primary afferent input (Ogbonna et al., 2013). Therefore, immunohistochemical analysis of microglial response in the spinal cord 28 days after MIA administration was performed. Intra‐articular injection of MIA in WT mice was associated with a significant increase in IBA1 expressing microglia within the ipsilateral dorsal horn compared to their respective saline controls (Fig. 5A–B and E). However, TASTPM mice injected with MIA did not display any change in the number of IBA1 labelled cells in the ipsilateral dorsal horn compared to their respective saline control mice (Fig. 5C–D and E). Furthermore, phosphorylated p38 in IBA1‐immunopositive microglia was significantly higher in ipsilateral dorsal horn of MIA‐compared to saline‐treated WT mice, with no alteration evident in TASTPM (Fig. 5A–D and F). The MIA‐injected TASTPM exhibit a trend to lower frequency of p‐p38 in IBA1 immunopositive cells in the ipsilateral dorsal horn, although not significant. As expected, no contralateral changes were observed in either WT or TASTPM spinal cords (Fig. 5E and F) as significant microgliosis in the spinal cord is only observed in spinal cords of 12‐month‐old TASTPM mice (Aman et al., 2016).

### 3.6 Increased opioidergic activity underlying impaired MIA‐induced mechanical hypersensitivity in TASTPM

**Figure 6:**
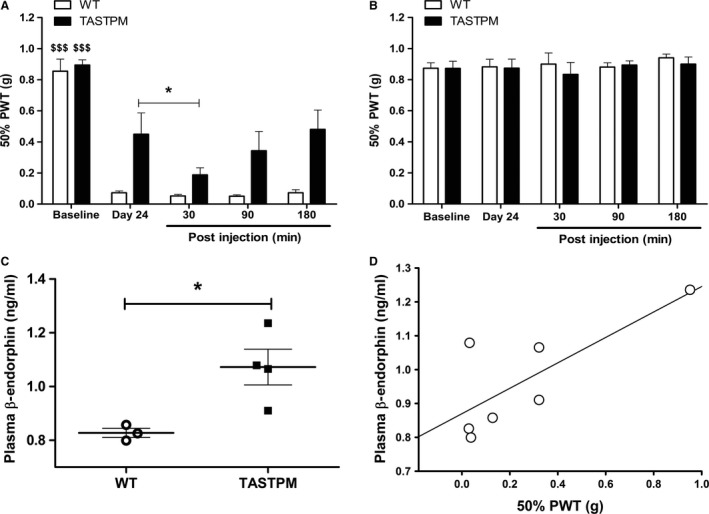
Increased opioidergic activity underlying impaired MIA‐induced mechanical hypersensitivity in TASTPM. Mechanical withdrawal responses of MIA‐injected mice were assessed in both hind paws, ipsilateral (A) and contralateral (B), prior to MIA injection (baseline). Twenty‐four days post‐MIA administration, mice were administrated (i.p.) with naloxone hydrochloride (1 mg/kg) and withdrawal thresholds were assessed in both hind paws at regular intervals prior to (Day 24) and up to 180 min post‐naloxone injection. Ipsilateral hind paw 50% paw withdrawal thresholds (PWT) on day 24 were significantly lower than respective baseline in both genotypes at all time points (^$$$^ *p* < 0.001). Naloxone hydrochloride injection reduced ipsilateral mechanical thresholds only in the TASTPM ipsilateral hind paw 30 min post‐administration (*p < 0.05) compared to pre‐injection values. No effect of naloxone on mechanical thresholds was detected in wild‐type (WT) ipsilateral (A). Also naloxone did not have any effect on the contralateral hind paws of both genotypes at any time point (B). Statistical analysis was conducted using two‐way repeated measures ANOVA followed by the Student–Newman–Keuls post hoc test. The 50% PWT values are presented as mean ± SEM (*n* = 6 (three males and three females) mice per group). Plasma concentration of β‐endorphin was determined by EIA 28 days post‐administration of MIA into the left knee joint of WT (one male and two females) and TASTPM (two males and two females) (C). MIA‐injected TASTPM displayed significantly greater levels of plasma β‐endorphin compared to WT (*p < 0.05, Student's *t*‐test). Data are presented as mean ± SEM (*n* = 3–4 per experimental group). A positive relationship between the level of plasma concentration of β‐endorphin and ipsilateral 50% PWT 28 days post‐MIA injection was detected using linear regression (*R* ^2^ = 0.589, *p* < 0.05) (D). Data plotted as individual animal plasma β‐endorphin and 50% PWT observed on day 28.

MIA‐induced mechanical hypersensitivity was evident in TASTPM which partially recovered during the late phase of the disease. Having established previously (Aman et al., 2016) that elevated opioidergic tone contributes to reduced nociceptive sensitivity to acute noxious stimulation in TASTPM mice, we examined the contribution of this system in impairment of chronic pain‐like behaviour. Systemic administration of naloxone, an opioid antagonist, unmasked the recovery of mechanical hypersensitivity in TASTPM as illustrated by a significant decrease in mechanical threshold values to comparable levels of that in WT mice 3 weeks after MIA administration (Fig. 6A). Naloxone did not affect the mechanical withdrawal thresholds in the contralateral hind paw of WT and TASTPM mice (Fig. 6B). Biochemical EIA analysis of the opioid peptide, β‐endorphin, revealed significantly elevated plasma levels in the TASTPM mice compared to WT 4 weeks after intra‐articular MIA injection (Fig. 6C). Moreover, we were able to demonstrate a significant positive relationship between the ipsilateral mechanical paw withdrawal thresholds on day 28 with the plasma concentrations of β‐endorphin (*R* ^2^ = 0.589, p < 0.05) (Fig. 6D). Hence, these data reinforce the notion that an increased endogenous opioidergic tone may underlie the altered behaviour response to chronic OA pain in the TASTPM model of AD.

### 3.7 Altered analgesic responses in TASTPM following MIA administration

Reversal of mechanical hypersensitivity was performed between day 0–day 7 and day 10–day 28 to represent the early/inflammatory and established late stage of the disease respectively. As weight bearing asymmetry was absent in the MIA‐injected transgenic TASTPM mice, we considered robust, and therefore only examined, reversal of mechanical hypersensitivity as a measure of analgesia.

**Figure 7:**
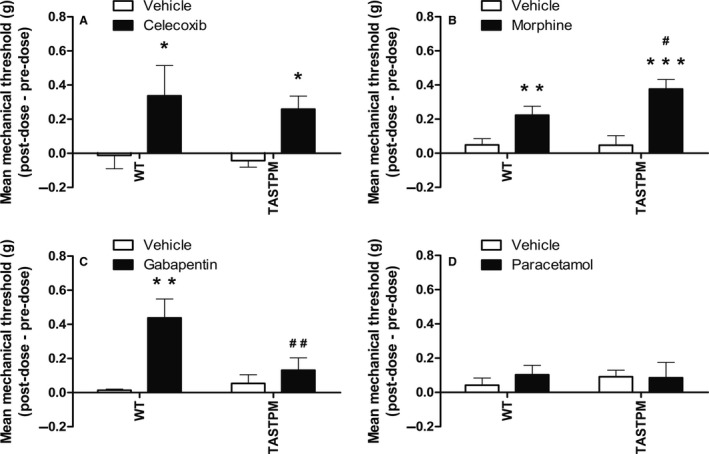
Altered analgesic responses in TASTPM Mice. Mechanical withdrawal thresholds of MIA‐injected mice were assessed prior to and at specified intervals post‐analgesic or vehicle administration. Both wild‐type (WT: three males and three females per treatment group) and TASTPM (four males and four females per treatment group) mice exhibited increase in mechanical thresholds 1 h post‐oral (p.o.) administration of celecoxib (30 mg/kg) on 6–7 days post‐MIA injection compared to their respective vehicle (methyl cellulose) controls (*p < 0.05) (A). Administration of morphine (6 mg/kg; s.c.), an opioid agonist, on 28 days after MIA injection resulted in significant reversal of mechanical hypersensitivity in both WT (4 males and 4 females per treatment group) and TASTPM (five males and five females per treatment group) mice (1 h post‐injection; **p < 0.01; ***p < 0.001) compared to their respective vehicle (saline) controls, with greater degree of analgesia observed in TASTPM compared to WT mice (^#^ *p* < 0.05) (B). Gabapentin (60 mg/kg; p.o.; 22–24 days post‐MIA) was only effective in reversing MIA‐induced mechanical hypersensitivity in WT (five males and three females per treatment group) mice (**p < 0.01; 3 h post‐injection), with no effect detected in TASTPM (five males and four females per treatment group) (*p* > 0.05), compared to their respective vehicle (dH_2_O) controls. The analgesic effect of gabapentin was significantly greater in WT mice compared to TASTPM (^##^ *p* < 0.01) (C). Paracetamol (300 mg/kg; p.o.) was ineffective in alleviating MIA‐induced mechanical hypersensitivity (*p* > 0.05) in both WT (Vehicle: three males and three females; Paracetamol: three male and two females) and TASTPM (Vehicle: two male and three females: Paracetamol: five males and four females) mice when administrated 28 days after MIA injection (30 min post‐injection), compared to their respective vehicle (saline) control group (D). Statistical analysis was conducted using two‐way ANOVA followed by the Student–Newman–Keuls post hoc test. Data are presented as difference between post‐dose and pre‐dose 50% PWT values that are illustrated as mean ± SEM (*n* = 5–10 mice per experimental group).

Celecoxib, a NSAID, was demonstrated to induce similar level of analgesia in both the WT and TASTPM mice during the early inflammatory phase (WT: 37% reversal of mechanical hypersensitivity relative to pre‐injection; TASTPM: 44%) (Fig. 7A). During the late established phase, the opioid agonist, morphine, induced greater level of analgesia in the TASTPM mice (WT: 24%; TASTPM: 59%) (Fig. 7B), whilst, during the same phase gabapentin, an anti‐epileptic drug failed to have any analgesic effect in TASTPM (WT: 40%; TASTPM: 8%) (Fig. 7C). Interestingly, paracetamol (WT: 10.9% ± 5.7%; TASTPM: 21.9% ± 10.4%) failed to induce analgesia in MIA‐injected WT and TASTPM mice during the established phase of OA pain (Fig. 7D).

Collectively these data highlight that drugs that induce analgesia in MIA‐injected WT mice do not necessarily have the same impact in the TASTPM model of AD, therefore indicating towards a possible alteration in nociceptive processing in chronic OA pain in the model of AD. Specifically, these results suggest that there is an attenuated pain‐like behaviour in response to MIA‐induced OA pain exhibited by the TASTPM transgenic mouse model of AD, which correlates with disruption in the opioidergic system in parallel with blunted central sensitization.

## 4 Discussion

In this study, we report an absence of MIA‐induced weight asymmetry and partial recovery of mechanical hypersensitivity in the TASTPM mouse model of AD. Histological and biochemical analysis revealed elevated opioidergic tone in parallel with diminished MIA‐induced microgliosis in the TASTPM spinal dorsal horn, despite similar knee joint pathology in comparison to MIA‐injected WT mice. This highlights a level of discrepancy between joint pathology and presentation of pain as clinical symptoms in OA (Hannan et al., 2000).

Furthermore, administration of NSAID attenuated mechanical hypersensitivity in TASTPM as effectively as in WT. However, changes in analgesic response during the established phase of MIA‐induced OA pain were illustrated by increased analgesic effect of morphine but an inability of gabapentin to attenuate mechanical hypersensitivity in the transgenic TASTPM mice. Therefore, our results provide support for disruption in the opioidergic system and contribution of central plasticity mechanisms to OA pain for the attenuated persistent pain‐like behaviour exhibited by TASTPM mice.

The MIA model of OA recapitulates multiple components of human OA disease progression, symptoms and associated pathology (Gregory et al., 2013). As expected, an intra‐articular injection of MIA induced immediate onset of mechanical allodynia and weight asymmetry in WT mice (Ogbonna et al., 2013). These behavioural outcome measures are a representation of referred allodynia and ongoing pain, respectively. In contrast, TASTPM mice did not display any sign of weight bearing asymmetry and exhibited a partial recovery of mechanical hypersensitivity from 14 days following administration of MIA, suggesting possible difference in the mechanisms underlying spontaneous and evoked pain in the TASTPM mice. However, mechanical hypersensitivity at 3 days after MIA injection was comparable in TASTPM and WT mice. This observation is in line with our previous findings that show the development of hypersensitivity in the carrageenan model of peripheral pain does not differ between WT and TASTPM mice (Aman et al., 2016).

Despite these behavioural observations, knee joint pathology in the form of degradation of articular cartilage accompanied by inflammation in the synovial membrane was exhibited by both the TASTPM and WT mice 4 weeks post‐MIA administration. Intra‐articular administration of MIA has been shown to display mechanical hypersensitivity in the absence of weight bearing deficits in a dose‐dependent manner, despite the knee joint pathologies (Ogbonna et al., 2013; Nwosu et al., 2016). Therefore, mechanical hypersensitivity and absence of ongoing pain exhibited by the transgenic model of AD may suggest a possible alteration of central plasticity mechanisms of MIA‐induced OA pain.

In fact, immunohistochemical analysis revealed the lack of spinal microglial response exhibited by MIA‐injected TASTPM mice in comparison to WT. Increase in spinal microglia is known to play an important role in central sensitization, as attenuation of microglial activation has been shown to correlate with reduced pain‐like behaviours in the neuropathic (partial nerve ligation) and inflammatory (zymosan) models of chronic pain (Clark et al., 2007; Staniland et al., 2010). Microglia activation in the spinal cord is known to play a critical component of chronic pain development. Spinal microgliosis (IBA1 immunopositive cells that also express p‐p38) in the dorsal horn correlated with decrease in grip force strength and increase in mechanical hypersensitivity in models of OA and rheumatoid arthritis, respectively (Lee et al., 2011; Clark et al., 2012; Nieto et al., 2016). Thus, diminished spinal microgliosis in TASTPM mice supports the notion that an alteration in the central spinal mechanisms may contribute to the alteration in persistent pain‐like behaviour exhibited by this model of AD.

In addition, administration of the anti‐epileptic drug gabapentin resulted in the reversal of MIA‐induced mechanical allodynia in only the WT group, as expected (Fernihough et al., 2004; Ivanavicius et al., 2007). In contrast, it failed to have any impact in MIA‐injected TASTPM mice. Gabapentin has been widely reported for treatment of neuropathic pain, with some evidence in OA (Rosner et al., 1996; Epstein and Childers, 1998). Elevated α2δ1 subunit mRNA expression in the DRGs and dorsal spinal cord of animal models of neuropathic pain (spinal nerve ligation) and OA (MIA) has been reported (Rahman et al., 2009; Zhou and Luo, 2014). Gabapentin is likely to modulate the activity of voltage‐gated calcium channels, probably through its ability to bind to α2δ1 subunit. As a result, this leads to a decrease in the neurotransmitter release and propagation of action potential in nociceptive neurons which in turn dampens nociceptive neuronal hyper‐excitability (Hayashida et al., 2008; Ossipov et al., 2010; Kukkar et al., 2013). These findings coupled with diminished spinal microgliosis in the transgenic TASTPM mice following MIA injection suggest a possibility of blunted spinal central sensitization, which may underlie the impaired persistent pain‐like behaviour exhibited by the TASTPM model of AD.

Furthermore, biochemical and pharmacological assessment indicated that the endogenous opioid may underlie the partial recovery of mechanical hypersensitivity in TASTPM following MIA administration, as illustrated by an increase in plasma β‐endorphin concentration and the ability of systemic administration of opioid receptor antagonist naloxone to unmask MIA‐induced mechanical hypersensitivity. Endogenous opioids may block nociceptive transmission via multiple mechanisms including actions on the mu opioid receptors located in the periphery, spinal cord and the brain (cortical and sub‐cortical regions) (Stein et al., 2003). Synthesis of β‐endorphin is carried out primarily in the anterior pituitary gland from its precursor protein proopiomelanocortin (POMC). It can be regulated by the hypothalamic–pituitary–adrenal axis through the corticotroponin‐releasing hormone (CRH) release from the hypothalamus in response to stress and/or pain, which signals for cleavage of POMC to generate β‐endorphin opioid peptides (Stein et al., 2003; Sprouse‐Blum et al., 2010). Increased CRH expression in AD parventricular nucleus has been associated with hyperactivity of the hypothalamic–pituitary–adrenal axis that may underlie the elevation of opioidergic peptide synthesis, as observed in the transgenic TASTPM mice (Raadsheer et al., 1995; Scherder et al., 2003). Moreover, a positive relationship between the level of plasma β‐endorphin and mechanical withdrawal thresholds 4 weeks after MIA suggests that elevated opioidergic tone in TASTPM mice may be responsible for the lack of ongoing pain and partial reversal of mechanical hypersensitivity (anti‐nociceptive) displayed post‐MIA.

Opioids (i.e. morphine) are used as an alternative for pain relief in OA patients who cannot use NSAIDs or paracetamol (Nuesch et al., 2009). In WT mice, our results are in agreement with previous reports where similar doses resulted in the reversal of MIA‐induced mechanical hyperalgesia and tactile allodynia (Combe et al., 2004; Fernihough et al., 2004). Surprisingly, the TASTPM mice not only exhibit an anti‐nociceptive effect of morphine but display a significantly heightened response in comparison to WT. Recent evidence has demonstrated greater prescription of transdermal opioids (i.e. buprenorphine and fentanyl) and long‐term usage in individuals with AD, despite significantly lower use of opioid amongst these individuals compared to cognitively intact controls. The underlying reason may be difficulty in ingestion of oral medicine in moderate to severe AD individuals, higher risk of mortality and/or the transdermal opioid scheme provides with greater ease as they need to be replaced after 3–7 days (Achterberg et al., 2013; Hamina et al., 2017). However, the underlying mechanism for this observation remains elusive owing to the increased opioidergic tone detected in the transgenic model of AD, which would suggest a reduced sensitivity to exogenous opioid agonist, morphine (Smith and Yancey, 2003). Therefore, these results implicate a possible regulation of the opioidergic system in AD.

In addition, celecoxib reduced mechanical allodynia when administrated on day 6–7 following MIA injection in both TASTPM and WT groups, suggesting an early component of inflammatory pain in this chemically induced model (Fernihough et al., 2004; Beyreuther et al., 2007; Ferreira‐Gomes et al., 2012; Rashid et al., 2013). These results provide evidence to support the notion that NSAIDs are as effective in inducing reversal of MIA‐induced mechanical allodynia in TASTPM as in the WT group, thus implicating that the early peripheral mechanisms, in particular the inflammatory aspect of MIA administration in the knee joint driving increased spinal nociceptive input resulting in pain, may be shared in the WT and this model of AD.

Moreover, paracetamol is often prescribed to patients for long‐term treatment (mild to moderate pain) where the adverse effects of NSAIDs raise concerns (Towheed et al., 2006). As expected, administration of paracetamol on day 28 post‐MIA failed to reverse mechanical allodynia in WT mice (Fernihough et al., 2004), thus indicating diminished analgesic potency of paracetamol in late and established phases of MIA‐induced OA. In the present study, we provide additional evidence in a model of AD, which also exhibited similar response to paracetamol as the non‐transgenic WT group, therefore suggesting that paracetamol on its own may not be an effective pain management option in both individuals with AD and cognitively intact patients at advanced stages of OA. This raises further concerns as relatively greater prescription and/or use of paracetamol have recently been reported in individuals with cognitive impairment for analgesia in comparison to cognitively intact individuals, which may reflect clinical difficulties in assessment of pain in such population (Haasum et al., 2011; Achterberg et al., 2013; Sandvik et al., 2014, 2016).

In summary, systemic changes in concordance with changes within the central mechanisms of pain processing in this model of AD resulted in differential effectiveness of analgesics, in particular opioids, during the course of OA development and progression. However, we cannot exclude the peripheral component from playing some roles and specific studies are required to address this possibility. Therefore, our data highlight the need to re‐evaluate current treatments, such as opioids, or develop novel therapeutic strategies for management of pain in individuals with AD.

## Author contributions

Y. Aman and M. Malcangio designed the experiments and drafted the manuscript. C. Ballard assisted with the design of experiments and review of manuscript. Y. Aman performed the experiments and analysed the data. T. Pitcher assisted with drug delivery. All authors discussed the results and commented on the manuscript.
